# Analysis of single nucleotide polymorphisms based on RNA sequencing data of diverse bio-geographical accessions in barley

**DOI:** 10.1038/srep33199

**Published:** 2016-09-12

**Authors:** Kotaro Takahagi, Yukiko Uehara-Yamaguchi, Takuhiro Yoshida, Tetsuya Sakurai, Kazuo Shinozaki, Keiichi Mochida, Daisuke Saisho

**Affiliations:** 1 Graduate School of Nanobioscience, Yokohama City University, 22-2 Seto, Kanazawa-ku, Yokohama, Kanagawa 236-0027, Japan; 2 Kihara Institute for Biological Research, Yokohama City University, 641-12 Maioka-cho, Totsuka-ku, Yokohama, Kanagawa 244-0813, Japan; 3 Cellulose Production Research Team, Biomass Engineering Research Division,RIKEN Center for Sustainable Resource Science, 1-7-22 Suehiro-cho, Tsurumi-ku, Yokohama, Kanagawa 230-0045, Japan; 4 Integrated Genome Informatics Research Unit, RIKEN Center for Sustainable Resource Science, 1-7-22 Suehiro-cho, Tsurumi-ku, Yokohama, Kanagawa 230-0045, Japan; 5 Faculty of Agriculture and Marine Science, Kochi University, 200 Otsu, Monobe, Nankoku, Kochi, 783-8502, Japan; 6 Gene Discovery Research Group, RIKEN Center for Sustainable Resource Science, 1-7-22 Suehiro-cho, Tsurumi-ku, Yokohama, Kanagawa 230-0045, Japan; 7 Biomass Research Platform Team, Biomass Engineering Research Division, RIKEN Center for Sustainable Resource Science, 1-7-22 Suehiro-cho, Tsurumi-ku, Yokohama, Kanagawa 230-0045, Japan; 8 Institute of Plant Science and Resources, Okayama University, Chuo 2-20-1, Kurashiki, 710-0046 Okayama, Japan

## Abstract

Barley is one of the founder crops of Old world agriculture and has become the fourth most important cereal worldwide. Information on genome-scale DNA polymorphisms allows elucidating the evolutionary history behind domestication, as well as discovering and isolating useful genes for molecular breeding. Deep transcriptome sequencing enables the exploration of sequence variations in transcribed sequences; such analysis is particularly useful for species with large and complex genomes, such as barley. In this study, we performed RNA sequencing of 20 barley accessions, comprising representatives of several biogeographic regions and a wild ancestor. We identified 38,729 to 79,949 SNPs in the 19 domesticated accessions and 55,403 SNPs in the wild barley and revealed their genome-wide distribution using a reference genome. Genome-scale comparisons among accessions showed a clear differentiation between oriental and occidental barley populations. The results based on population structure analyses provide genome-scale properties of sub-populations grouped to oriental, occidental and marginal groups in barley. Our findings suggest that the oriental population of domesticated barley has genomic variations distinct from those in occidental groups, which might have contributed to barley’s domestication.

Food security is an urgent global issue for ensuring sustainable food supply for the current and future generations. Climate change adversely affects the global agricultural system, and thus increases the risk of food shortage^1^,^2^. Mining and biodiversity utilisation should be a potential promising strategy to prevent declines in crop production; this involves the systematic domestication of newly designed crops that have improved productivity^3^.

Barley (*Hordeum vulgare* ssp. *vulgare*) is the fourth most important cereal crop worldwide following wheat, rice, and maize (https://www.croptrust.org/crop/barley/). It is believed to be among the oldest crop species in the world and it was domesticated from the large-seeded wild barley (*H. vulgare* ssp. *spontaneum*)^4^,^5^. Archaeological evidences indicate that the seed size of wheat and barley began to increase during Pre-Pottery Neolithic A and early Pre-Pottery Neolithic B, dating approximately from 11,100 to 10,500 before present, at the sites Jerf el Ahmar in Syria^6^ and ZAD-2 in Jordan^7^,^8^. In many crops, including barley, multiple domestications have been hypothesised^9^,^10^,^11^,^12^,^13^,^14^,^15^, as multiple progenitor populations lead to numerous independent domestication processes with complex demographic histories^16^. In multiple domestication, many agronomical important mutations of independent origins could contribute simultaneously to the domestication processes^17^.

As barley is one of the most widely adapted crops, barley germplasm pools might possess genetic diversity associated with adaptability to various environmental conditions. They should be able to be used as a source for mining valuable allelic variations to improve barley productivity. The large *ex situ* barley seed collections allow the exploration of useful genes and allelic variations that can contribute to the improvement of productivity and sustainability of barley. Several studies investigated the extent of genetic variations and population structure of wild barley by using different seed collections^18^,^19^. Phylogeographical analysis performed using 318 wild barley accessions from the Wild Barley Diversity Collection suggested that the primary structured population differentiated into West and East populations across Zagros Mountains^20^. In domesticated barley, the population structure analysis based on five nuclear loci from >250 accessions showed a primary bisected population structure, which mainly represented a cluster consisting of landraces from Europe/North Africa, Ethiopia, and the Near East (occidental) and another cluster with landraces from Asia (oriental)^21^. This genetically differentiated pattern of domesticated barley was well correlated with the heritage research reported previously^22^. The Asian group was designated as “Oriental” barley whereas the “Occidental” group is distributed in the western part of Eurasia and North Africa. During the multiple domestications of barley, the eastern wild barley ancestors could have greatly contributed to the differentiation of Asian landraces, which indicates that valuable genes and allelic variations are involved in the adaptive traits of varieties to local environmental conditions in Asia^11^,^23^,^24^. Therefore, mining of genetic diversity from the Asian landraces might allow the detection of valuable variations that can be used to improve the adaptability of barley against various environmental conditions as well as improve our understanding on the evolutional history of this crop.

In-depth analysis of genes and alleles involved in crop domestication and adaptability to environments requires the use of comparative re-sequencing analyses and high-density single nucleotide polymorphism (SNP) genotyping^25^,^26^,^27^. In barley, genome-wide SNPs among accessions were identified using Sanger sequencing of cDNAs and pyrosequencing of genomic DNA using the genomic reduction method^28^,^29^. More recently, the array-based genotyping platform Infinium iSelect, consisting of 7,842 SNPs, was used to genotype 2,417 barley accessions sampled from the 33,176 accessions comprising the USDA National Small Grains Collection to assess their genetic diversity and population structure^30^. Although chip- or array-based genotyping has enabled the analysis of thousands of polymorphisms that were validated in advance, these methods involve the inclusion of ascertainment biases caused during the polymorphism discovery process^31^,^32^. The ascertainment biases can be avoided performing direct whole-genome re-sequencing, RNA sequencing (RNA-seq), or exome-sequencing using diverse panels of accessions to assess the genetic diversity in a germplasm set more accurately^33^,^34^. An exome capture kit with a 90.2 Mb capture space was designed for barley and applied to genotype three *H. spontaneum* and 13 *H. vulgare* accessions. The phylogenetic relationships of these barley accessions were obtained based on 122,940 bi-allelic SNPs identified using exome-sequencing analysis^33^. RNA-seq is a useful method to rapidly acquire comprehensive gene expression profiles and to generate genome-scale datasets of sequence polymorphisms^35^. Scalability and cost-effectiveness of RNA-seq methods allow in-depth analysis of polymorphisms found in the exome regions even in a species with a large and complex genome, such as barley.

In this study, to assess the genetic diversity of oriental barley landraces, we performed RNA-seq analysis of 19 diverse accessions of domesticated barley and one wild barley variety, *H. vulgare* ssp. *spontaneum* H602. Based on RNA-seq results, we obtained the global distribution of SNPs on the transcribed regions of the several barley landraces, including Asian barley. Using this SNP dataset, we determined the phylogenetic relationships among oriental and occidental barley landraces and wild barley. Furthermore, using this SNP dataset generated from RNA-seq and another generated using a publicly available exome-sequencing dataset provided by Mascher *et al*.^33^, we estimated the population structure of domesticated barley accessions. Based on the entire barley population, we revealed domesticated barley genomic diversity and population subdivision in a genomic context.

## Results and Discussion

### Transcriptome sequencing and transcribed region annotation

**Figure 1:**
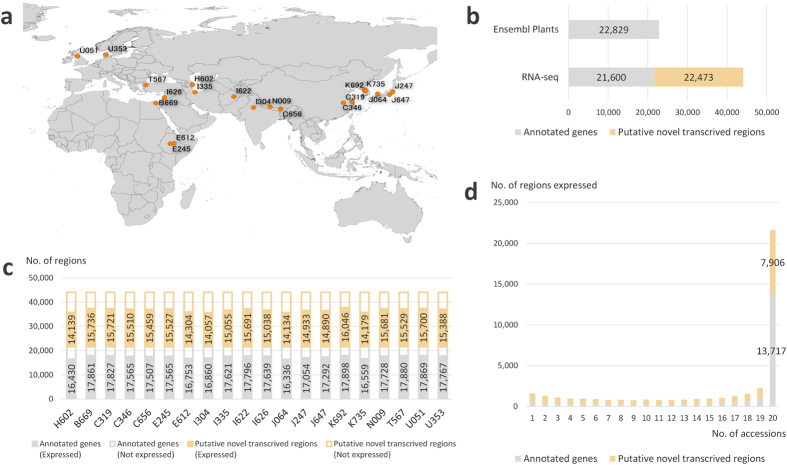
Barley accessions and transcribed regions identified using RNA-seq analysis. (**a**) Geographic origin of the barley accessions used in this study. The origins were plotted on a map data made with Natural Earth (http://www.naturalearthdata.com/) through the QGIS 2.8.3 Wein interface (http://qgis.org/en/site/index.html). (**b**) Number of transcribed regions identified using RNA-seq analysis, which were compared to those annotated using the Morex genome in Ensembl Plants. (**c**) Number of transcribed regions expressed in each accession (FPKM ≥ 1). (**d**) Range of regions expressed across the 20 barley accessions.

We selected 19 domesticated barley accessions (18 landraces and an improved variety) of several biogeographical origins, based on a previous analysis of molecular phylogenetics and growth habits^21^,^36^. The properties of these barley accessions are summarised in Supplementary Table 1. In addition, we used an ancestral variant of wild barley, H602, which is widely used as a reference species of *H. spontaneum*. The geographic origin of each barley accession is shown in Fig. 1a. Further, *H. vulgare* L. ‘Morex’ was used as a reference to construct the genomic framework for barley’s RNA-seq analysis.

To rapidly generate genome-scale SNPs datasets for each barley accession from the transcribed regions, we sequenced mRNAs from the leaves and roots. Using an Ion Proton semiconductor sequencer based on the pyrosequencing method, we generated 278 million reads and mapped more than 94% (263 million reads) of these reads to the pseudomolecules of the Morex genome (Table 1). Using the gene annotation performed for Morex available from the Ensembl plants website (http://plants.ensembl.org/index.html), we predicted gene structure based on RNA-seq reads mapping to the Morex genome using Cufflinks. Comparing our gene structural annotation to that of Morex, 94.6% of the annotated genes had corresponding sequence reads and 22,473 newly identified transcribed regions showed sequence similarity to the protein sequences of at least one grass species (Fig. 1b). From 7,700 to 12,000 protein sequences showed sequence similarity to barley’s novel potentially transcribed regions (Supplementary Figure 1). These results suggested that a large number of transcriptional units (TUs) were not annotated in the current Morex genome annotation, and that the identification of such novel TUs might be facilitated using our RNA-seq reads. Comparative analysis between the transcripts of *Brachypodium*, a Pooideae model grass, and the Morex genome showed that many sequences expressed in *Brachypodium* were mapped to the inter-genic regions of the Morex genome^37^. Mascher *et al*.^33^ performed whole exome-sequencing of barley and conducted gene structural prediction by using RNA-seq contigs to identify as many TUs as possible^33^. Results of the present study suggest that comprehensive transcriptome sequencing and comparative analyses with related species are essential to further annotate TUs in the barley genome, which might provide a genomic foundation to explore genetic polymorphisms in the genic regions.

We assessed the number of regions that are transcribed in each of the 20 barley accessions. In each accession, we analysed 30–33 K regions that showed expression levels measured as fragments per kilobase of exon per million mapped fragments (FPKM) values ≥1, which included about 16–17 K annotated genes and 14–16 K putative novel transcribed regions (Fig. 1c). We also investigated the levels of gene expression in the 20 barley accessions and found that more than 13 K annotated genes and 7 K putative novel transcribed regions were commonly expressed in all the 20 accessions (FPKM ≥ 1; Fig. 1d). Therefore, RNA-seq analysis appears to be a feasible approach to compare transcribed regions among accessions, and to allow a rapid and comprehensive acquisition of SNP data from accessions, without requiring pre-designed tools such as sequence capture probes or primer sets for targeted re-sequencing. For barley, in particular, that has a large genome and immature gene-structure annotation, RNA-seq analysis of several accessions might allow accumulating genome sequence variations to elucidate on the diversity of the population.

### SNP discovery and quality control

Mapping RNA-seq reads into the genomic framework of Morex allowed identifying SNPs using the following programs: freebayes, glfMultiples, and samtools (Table 2). Although each program yielded a distinct number of SNPs, we extracted those that were commonly called by all programs, as these were considered the accurate SNPs^38^ (Table 2). Some studies have recently reported a significant difference between the set of variants called by different variant callers^39^,^40^. In our study, we also observed changes in the ranks of the accessions among the different programs, which suggests that the trend in variant calling is not only determined by a simple threshold but also by factors such as the sequencing method, sequencing depth, or quality of the reference sequences^41^, as well as calling argorithums and mathematical models^42^.

**Figure 2:**
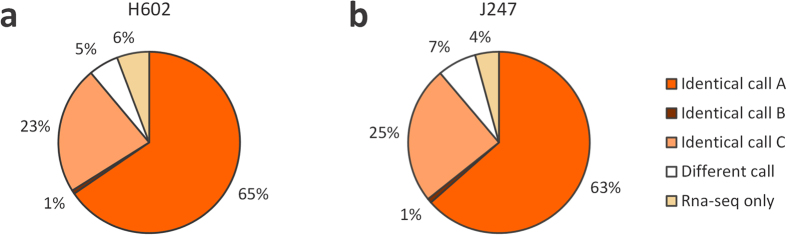
Accuracy assessment of the RNA-seq-based SNPs and its comparison with exome-sequencing-based SNPs. Proportion of RNA-seq-based SNPs also present in the exome-sequencing data obtained by Mascher *et al*.^33^ in H602 (**a**) and in J247 (**b**). Identical call A: SNPs consistent with exome-sequencing-based SNPs and called by all programs. Identical call B: SNPs consistent with exome-sequencing-based SNPs and called by one or two program(s). Identical call C: SNPs shared with raw data of the exome-sequencing dataset, which were filtered by our threshold. Different call: SNPs inconsistent between the RNA-seq and the exome-sequencing datasets. RNA-seq only: SNPs only observed in the RNA-seq dataset.

Our RNA-seq analysis and the exome-sequencing analysis performed by Mascher *et al*.^33^ enabled comparing the SNP datasets of H602 (OUH602) and J247 (Haruna Nijo), which were common to both studies. We applied the three programs to call SNPs in these exome-sequencing datasets, and identified 355,980 and 268,235 SNPs that were commonly called in the SNP call programs from H602 and J247, respectively (Table S2). Therefore, we assessed the accuracy of the RNA-seq-based SNPs in comparison with those from the exome-sequencing analysis. We also compared the 55,403 and 38,729 SNPs obtained for H602 and J247, respectively (Fig. 2, Table 2). Overall, 65% and 63% of the SNPs found for H602 and J247, respectively, in our RNA-seq analysis were also identified in the exome-sequencing analysis data, without ambiguity (Fig. 2. Identical call A: SNPs identically called by the three programs in both the RNA-seq and the exome-sequencing analyses). Further, 1% of the SNPs found in the RNA-seq dataset in H602 and J247 were shared by the exome-sequencing dataset (Fig. 2. Identical call B: SNPs identically called by the three programs in the RNA-seq and by one or two program(s) in the exome sequencing dataset). Twenty-three percent and 25% of the SNPs called for H602 and J247, respectively, were consistent with the raw dataset of SNP calls based on exome-sequencing (Fig. 2. Identical call C), which were filtered according to our threshold (see Methods). Among the RNA-seq-based SNPs, 5% in H602 and 7% in J247 were inconsistent with those in the exome-sequencing (Fig. 2. Different call). Of the remaining RNA-seq-based SNPs, 6% in H602 and 4% in J247 were only observed in RNA-seq (Fig. 2. RNA-seq only). Using the published exome-sequencing dataset, we could assess the accuracy of SNPs based on the RNA-seq analysis and determine the ideal combination of programs and thresholds for calling accurate SNPs. Our quality assessment suggested that intersecting the SNPs identified by the multiple calling programs provided further accurate SNPs with higher confidence. Table 2 summarises the number of SNPs identified based on RNA-seq analysis, ranging from 38.7 K (in J247) to 79.9 K (in I622) in each accession (Table 2).

Several methods are currently available to carry out the discovery and genotyping of genome-scale polymorphisms. These methods are mainly classified into pre-designed and *de novo* approaches: SNP chips and exome-sequencing are representatives of the pre-designed approach, which require hybridisation probes or PCR primers to enrich targeted regions. High-density SNP chips like Illumina’s^TM^ Infinium beads chip can support ~1 million SNPs in a custom designed panel. For exome-sequencing, the SeqCap EZ system (Roche), used for barley’s exome-sequencing, can cover up to 200 Mb of custom regions. If a high-quality and well-annotated reference genome is available, these pre-designed approaches allow genotyping validated SNPs and/or resequencing targeted regions with deeper coverage. On the other hand, whole genome re-sequencing, genotyping-by-sequencing (GBS), and RNA-seq are becoming popular approaches for *de novo* genotyping. Although recent price decline in sequencing cost has made it possible to perform whole genome re-sequencing in population analysis, it is still the most expensive approach, and shallow sequencing depth often becomes a problem, depending on the genome size of the target species. GBS is a cost effective approach for genome-scale genotyping, and has been widely used in several species, being feasible for large genomes such as those of wheat and barley. Although sequencing depth in GBS depends on the genome size of the target species, this method enables reducing genome representation, performing a cost-effective polymorphism discovery, and genotyping genic and intergenic regions. Because GBS does not always require a reference genome sequence, it can be used in population analysis and to construct an initial genetic map in organisms whose genome has not been sequenced. In this study, we applied RNA-seq to examine genome-scale polymorphisms among barley accessions. Although RNA-seq approach only covers SNPs on transcribed regions of expressed genes in analysed tissues, it is a cost-effective and well scalable method to analyse polymorphisms on expressed genes.

Although the range of SNPs identified in our RNA-seq analysis is lower than that obtained during deep exome-sequencing, it is similar or higher than those reported in previous GBS studies of Triticeae crops^43^,^44^. Such range of SNPs might be useful for genome-wide genotyping at middle-scale density, i.e. between amplicon-sequencing of panelled target genes and capture-based exome-sequencing densities. Our data suggested that sequencing a pooled RNAs sample is efficient to avoid tissue dependency and to increase the number of sequenced regions and identified SNPs. In addition, RNA-seq does not always require a reference genome, as a *de novo* transcriptome assembly might serve as proxy for a reference genome, enabling reducing genome representation without depending on its size and restriction sites. Therefore, RNA-seq and GBS appear to be reliable approaches to rapidly acquire genome-scale polymorphisms in organisms without reference genome information and mature structural annotation. RNA-seq might also be an efficient approach to rapidly acquire genome-scale polymorphism datasets at the population scale using a core-collection or sub-populations of landraces and mapping populations.

### SNP profiles of the 20 barley accessions

**Figure 3:**
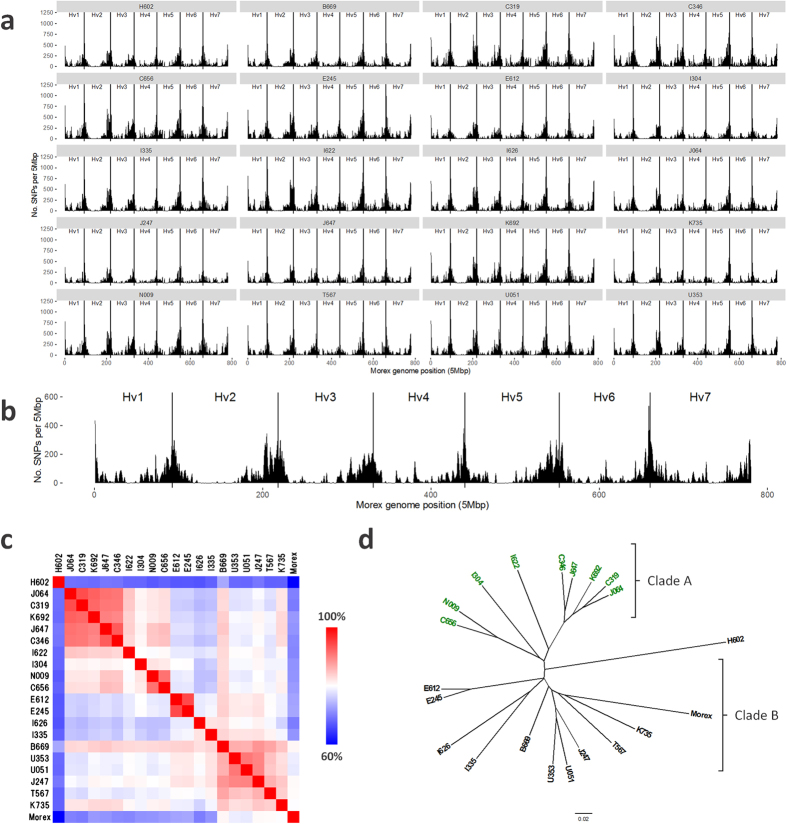
Genome-wide distribution of the SNPs identified using RNA-seq analysis of the 20 diverse barley accessions. (**a**) Distribution of the RNA-seq-based SNPs of the 20 barley accessions, using the Morex genome as a reference. The numbers of SNPs were computed for each chromosome using 5 million bases sliding windows. (**b**) Distribution of the bi-allelic RNA-seq-based SNPs. (**c**) Genome-wide sequence identity based on the bi-allelic SNP dataset across the 20 barley accessions and Morex. (**d**) Neighbour-joining tree of the 20 barley accessions and Morex based on the bi-allelic SNP dataset.

Using the SNP dataset described above, we identified the genome-wide SNP profiles of each barley accession (Fig. 3a). The density of SNPs was higher in the distal regions of each chromosome and was correlated with that of annotated genes in the barley genome. These profiles were in agreement with those determined using exome-sequencing^33^. For molecular phylogenetic analysis, we generated bi-allelic SNP datasets consisting of 37,774 SNPs with no missing data called from the 20 barley accessions and Morex (Fig. 3b).

To estimate the genetic diversity among the 20 accessions and Morex, we calculated the pairwise proportion of identical nucleotides at the bi-allelic SNP sites. We found that approximately 60% of the bi-allelic SNPs were conserved between the wild barley, H602, and each of the remaining domesticated barley accessions (Fig. 3c). Using the bi-allelic SNP datasets, we determined the phylogenetic relationships among the accessions. In the phylogenetic tree, H602 was allocated to a distinct outer branch, as expected (Fig. 3d). Asian accessions were divided into two major groups: A, formed by nine Asian accessions; and B, comprising two Asian accessions (K735 and J247) and non-Asian accessions. K735 was originally collected from the Korean peninsula (approximately 37° N, 127° E) and is classified as the Manchurian type, which contributed to the breeding of North American six-rowed malting variety Morex, whereas J247 is Japanese modern variety for malting, developed by crossing European malting barley cultivars^45^. Thus, the phylogenetic relationships based on genome-scale polymorphisms reflect the genomic history of these accessions dictated by their modern breeding process. The topology of the tree and phylogenetic positions of each accession were in agreement with the previously reported population structure of domesticated barley^21^.

### Barley molecular phylogeny based on genome-scale SNP datasets

**Figure 4:**
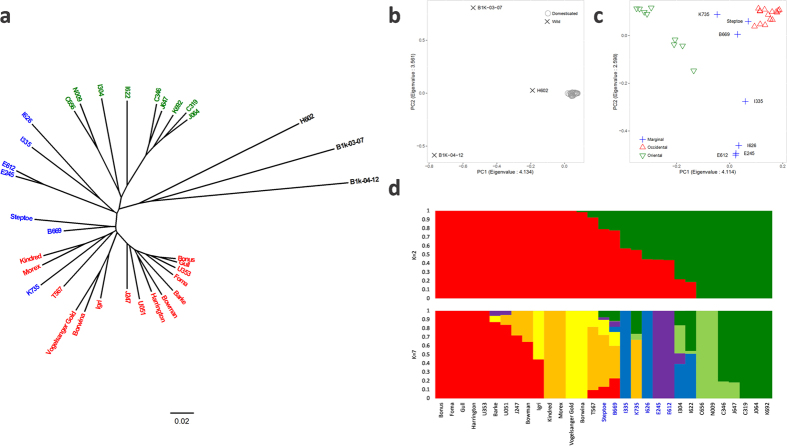
Molecular phylogeny and population structure according to the integrated SNPs dataset derived from RNA and exome-sequencing in barley. The domesticated barley accessions were classified into three groups based on geographic origins: occidental (red), oriental (green), and marginal (blue). (**a**) Neighbour-joining tree of 34 barley accessions based on the bi-allelic SNP dataset generated using the RNA-seq and exome-sequencing datasets (Mascher *et al*.^33^). (**b**) PCA plot of the 31 domesticated barley accessions and three wild barley accessions. (**c**) PCA plot of the 31 domesticated barley accessions. (**d**) Bayesian clustering of the 31 domesticated barley accessions using *K* = 2 (upper) and *K* = 7 (lower) populations.

To conduct an in-depth analysis of barley divergence, we integrated the SNPs obtained from RNA-seq and exome-sequencing. We used the RNA-seq dataset of 20 barley accessions (Table 2) as well as the exome-sequencing datasets of 13 barley accessions comprised from two spontaneous and 11 domesticated barley accessions (mainly categorised to improved variety) (Table S2). The bi-allelic SNP datasets consisting of 40,364 SNPs with no missing data were generated from 33 barley accessions and Morex. Based on the bi-allelic SNP datasets, we determined the phylogenetic relationships among accessions (Fig. 4a). Within the clade comprising the 31 domesticated barley varieties, accessions were primarily classified into two groups with some intermediates. The nine Asian accessions were clustered into one clade (Fig. 4a), which corresponded to clade A in Fig. 3d and represented oriental accessions sequenced in this study. The other domesticated barley accessions were clustered into another clade that comprised the accessions grouped in clade B of Fig. 3d and all the cultivated barley accessions used in previously sequenced by exome-sequencing(Fig. 4a). A principal component analysis (PCA) using the bi-allelic SNP datasets of the 34 barley accessions evidenced the agglomeration of the domesticated accessions, all of which differed from the three wild barley accessions (Fig. 4b). The PCA based on the 30,547 bi-allelic SNPs called from the domesticated barley accessions only, showed a clear separation between the oriental and occidental barley accessions along the first principal component (PC1), with coastal Mediterranean accessions (*n* = 7) appearing between them (Fig. 4c). The phylogenetic tree and the PCA plots suggested that the presumed oriental sub-populations of domesticated barley possess particular variations that are different from those in the other accessions. In addition to phylogenetic and principal components analyses, we also examined sub-population structure in domesticated barley using the Bayesian clustering method. The number of populations (*K*) indicated by the delta *K* values^46^ the best-fitted model was two (Supplementary Figure 2). In this model, oriental and occidental barley were clearly classified into distinct clusters and the probability scores of some accessions suggested an admixture pattern (Fig. 4d). We also examined the model showing the second highest delta *K* value (*K* = 7) to elucidate the population structure of the domesticated barley accessions in a further classified model (Fig. 4d), where oriental and occidental populations being subdivided into two and three clusters, respectively. Although the Ethiopian (E245 and E612) and Near East (I335 and I626) landraces formed marginal clusters in the *K* = 2 model, these accessions formed differentiated clusters in the *K* = 7 model. Steptoe, B669, and K735 accessions showed mixed membership probabilities toward oriental and occidental populations in both models and in the PCA were plotted between oriental and occidental accessions, along PC1 (Fig. 4c), suggesting they form a marginal group. Based on these results, we classified domesticated barley accessions into three groups: oriental (C319, C346, C656, I304, I622, J064, J647, K692, and N009), occidental (Barke, Bonus, Borwina, Bowman, Foma, Gull, Harrington, Igri, J247, Kindred, Morex, T567, U051, U353, and Vogelsanger Gold), and marginal (B669, E245, E612, I335, I626, K735, and Steptoe).

### Genomic diversity in domesticated barley sub-populations

**Figure 5:**
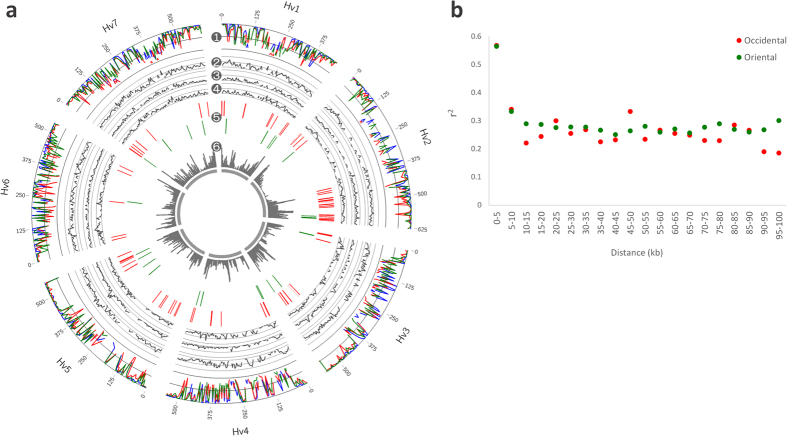
Overview of the genomic diversity in domesticated barley sub-populations. (**a**) Genome-scale statistics and LD blocks of domesticated barley sub-populations based on the bi-allelic SNP dataset. (1) Haplotype diversity calculated for the occidental (red), oriental (green), and marginal (blue) barley populations using sliding windows of 5 million bases. (2–4) Pairwise *F*_*ST*_ values calculated for the occidental and oriental groups (2), occidental and marginal groups (3) and oriental and marginal groups (4) using sliding windows of 5 million bases. (5) LD blocks (≥10 kb) of occidental (red) and oriental (green) barley sub-populations. (6) Density distribution of transcribed regions for the integrated dataset comprising annotated genes and the putative novel transcribed regions identified in the RNA-seq analysis. (**b**) LD decay plots for the occidental (red) and oriental (green) barley sub-populations. The average *r*^2^ values were plotted against physical distance.

To elucidate the genomic diversity of the domesticated barley populations, we calculated haplotype diversity and pairwise *F*_*ST*_^47^ using 5 million bases sliding windows for each of the three groups, i.e. oriental, occidental, and marginal. The marginal group showed higher average haplotype diversity than the oriental and occidental groups (Table 3). We also observed lower *F*_*ST*_ values between the occidental or oriental groups and the marginal group than between occidental and oriental groups (Table 3). These results indicated a greater divergence between oriental and occidental sub-populations than between any of them and the marginal group, suggesting each sub-population adapted to particular geographic properties throughout their demographic history. The genomic regions showing *F*_*ST*_ levels above the average level might have been the ones contributing the most to the divergence of domesticated barley sub-populations (Fig. 5a). We also estimated the linkage disequilibrium (LD) patterns of oriental and occidental sub-populations in a genome-scale. Oriental and occidental sub-populations showed LD decays between 5 and 10 kb (Fig. 5b), which were longer than that observed in maize (<1 kb)^48^ and *Arabidopsis thaliana* (L.) Heynh. (3–4 kb)^49^, but shorter than those of sorghum (10.3 kb for landraces and 19.7 kb for improved inbreds)^50^, rice (65 kb for *Oryza sativa indica* and 200 kb for *O. s. japonica*)^51^, and soybean (~75 kb and ~150 kb for wild and cultivated varieties, respectively)^52^. In contrast to the similar behaviour of the LD decays observed in the oriental and occidental sub-populations, distinct LD blocks were found among the differentiated barley groups (Fig. 5a). The results suggested that domesticated barley has selective genetic traits that contributed to the differences found between oriental and occidental accessions. Our RNA-seq based SNP analysis enabled us to identify SNPs on genic regions that are specific to oriental or occidental accessions (Table S3). Thus, these data might be useful to explore genomic regions and genes associated to particular traits in each sub-population, including the oriental and occidental accessions.

## Conclusions

The genetic patterns of differentiation of the Asian landraces of oriental barley might elucidate on the potential genetic adaptability of barley to the East Asian monsoon area. The genome-wide variation determined using the RNA-seq technology might become an established tool to address future food security issues. Our results suggest that oriental landraces and/or Asian barley might be important for the future increase of barley productivity.

## Materials and Methods

### Plant materials

Total RNA was extracted using the RNeasy plant mini kit (QIAGEN GmbH, Hilden, Germany) from the leaves and roots of 19 domesticated barley (*Hordeum vulgare*) accessions and one wild barley (*H. spontaneum*) accession, according to the manufacturer’s instruction. The accessions used were obtained from the domesticated barley collection preserved at the Institute of Plant Science and Resources, Okayama University, and from the publicly available Barley database (http://www.shigen.nig.ac.jp/barley). The identifiers, common name, and historical data of the barley accessions are summarised in Supplementary Table 1. The barley plants used for RNA extraction were grown using hydro-culture in a growth chamber. The growth chamber was maintained at 16-h light: 8-h darkness at 22 °C. First leaf blades (L1) and the remaining parts of shoots (L2), as well as roots (Rt), were separately harvested at 14 days after germination. The RNA extracted from L1, L2, and Rt from each barley accession was evenly pooled. All RNA samples were assessed using the Agilent 2100 bioanalyzer (Agilent Technologies, USA) using the Agilent RNA 6000 Pico Kit (Agilent Technologies, USA). Total RNAs were stored at −80 °C until library preparation for RNA sequencing.

### RNA sequencing

Poly(A) + RNAs were purified using MicroPoly(A)Purist™ Kit (Life Technologies, USA), according to the manufacturer’s instructions, and assessed using the Agilent 2100 bioanalyzer (Agilent Technologies, USA) and the Agilent RNA 6000 Pico Kit (Agilent Technologies, USA). Libraries for strand-specific RNA sequencing were obtained in an Ion torrent sequencer using the Ion Total RNA-Seq Kit v. 2 (Life Technologies, USA), according to the manufacturer’s instructions, and Ion Xpress Barcode Adapters (Life Technologies, USA) developed for each accession. Templates for the 200 base-read libraries sequenced using the Ion Proton semiconductor sequencer (Life Technologies, USA) were prepared using the Ion P1 Template OT2 200 Kit v. 3 (Life Technologies, USA), Ion P1 Sequencing 200 Kit v. 3 (Life Technologies, USA), and Ion P1 Chip Kit v. 3 (Life Technologies, USA), according to the manufacturer’s instructions. Sequencing was performed using an Ion Proton sequencer (Life Technologies, USA) equipped with an Ion P1 chip.

### Data analysis of RNA-seq reads

The pseudomolecular sequence of Morex was downloaded from Ensembl Plants FTP site (082214v1.25, ftp://ftp.ensemblgenomes.org/pub/plants/release-25/fasta/hordeum_vulgare/dna/) and used as a reference sequence to map the RNA-seq reads using the -n 16 -v -Y -u -o 2 stage1 map4 command in Tmap v. 3.4.1 (Life Technologies, USA). Binary Alignment/Map (BAM) files with mapped reads were generated using samtools^53^ and sorted BAM files were subjected to further analyses.

Putative novel transcribed regions were identified based on the RNA-seq data using the cufflinks and cuffmerge commands in the Cufflinks package v. 2.2.1^54^ and the Morex gene structural annotation dataset retrieved from the Ensembl Plants FTP site (082214 v1.25, ftp://ftp.ensemblgenomes.org/pub/plants/release-25/gff3/hordeum_vulgare/). The cufflinks command with –g option was used to identify putative novel transcribed regions based on the RNA-seq data of each accession, in addition to the known genes annotated in the Ensembl Plants dataset. The cuffmerge command was used to merge the newly identified transcribed regions with the known genes. To confirm the shared homology between the novel transcribed regions and other known genes, sequences ≥ 200 bp were compared to the deduced proteome datasets of some grass species (*Aegilops tauschii* Coss.*, Brachypodium distachyon* (L.) P. Beauv., *O. sativa, Setaria italica* (L.) P. Beauv.*, Sorghum bicolor* (L.) Moench*, Triticum aestivum* L.*, T. urartu* Thumanjan ex Gandilyan, and *Zea mays* L.) downloaded from Ensembl Plants FTP site (ftp://ftp.ensemblgenomes.org/pub/plants/release-30/fasta/), using the basic local alignment search tool and a translated nucleotide query (BLASTx, -e 1e -5). The cufflinks command with –G option was used to calculate FPKM values, based on the merged gene annotations including previously annotated genes and the putative novel transcribed regions for each accession. Regions with FPKM ≥ 1 were defined as expressed genes or regions.

### SNP discovery using RNA-seq reads

BAM files of each barley accession were subjected to SNP calling using freebayes v. 1.0.1–2 g0cb2697^55^, glfMultiples v. 2010-06-16^56^, and samtools v. 1.3, with default parameter settings. In each accession, the SNPs holding alternative allele frequency above 90% in depth and position read depth ≥5 were identified as putative homozygous SNPs. We omitted SNPs that were out of the threshold. The read depth at each locus was calculated using genomeCoberageBed in BEDTools v. 2.20.1^57^. The intersectional SNPs identified by each of SNP callers that fulfilled the thresholds of read depth and allele frequency were subjected to further analyses as a set of reliable SNPs.

### SNP discovery using public exome-sequencing reads

The exome-sequencing-based SNPs in barley^33^ (ERR271694-ERR271704, ERR271716-ERR271729, and ERR271735-ERR271737) were retrieved to compare them with the RNA-seq-based SNPs and integrate both SNPs datasets for further analyses. The exome-sequencing reads were trimmed using Trimmomatic v. 0.32 using the settings -thread 8 LEADING: 20 TRAILING: 20 SLIDINGWINDOW: 4:15 MINLEN: 36. The trimmed reads were mapped to the Morex pseudomolecule sequence using the BWA (mem) program v. 0.7.12-r1039^58^ with a parameter setting of -t 8. The uniquely mapped and properly paired reads were used in further analysis. Possible duplicated reads were removed using the rmdup command in samtools. The SNPs of the exome-sequencing data were identified using the same methods as in RNA-seq-based SNPs discovery.

### Bi-allelic SNP dataset construction

Bi-allelic SNP datasets were constructed based on the SNPs that met the following criteria: (i) The SNP was detected in at least one accession; (ii) read depth of the SNP position was≥ 5 in all accessions; and (iii) other nucleotides were found at the SNP position in <10% of all accessions.

### Population analyses

The phylogenetic relationships between barley accessions were determined through phylogenetic analyses, PCA, and Bayesian clustering using the bi-allelic SNP datasets. Neighbour-joining trees were generated using MEGA v. 6.0 with a p-distance model^59^. PCA was performed using smartpca in EIGENSOFT v. 6.0.1^60^ with default settings. PC values were plotted using R program v. 3.1.0. The population structure of the domesticated barley accessions was estimated in STRUCTURE v. 2.3.4^64^ using 30,000 burn-in times and 60,000 replications. The number of populations (*K*) was set from 1 to 15, with three runs for each *K* value. To detect the optimal size of *K*, delta *K* values were calculated using Structure Harvester on the web (http://taylor0.biology.ucla.edu/structureHarvester/).

### Haplotype diversity and *F*_*ST*_ calculation

The genomic diversity between domesticated barley sub-populations, haplotype diversity, and pairwise *F*_*ST*_ were calculated using the PopGenome package^62^ in R v. 3.1.0 with 5 million bases sliding windows. These values, along with the barley chromosomes, were represented on the Morex reference genome using circos v. 0.67–5^63^.

### Linkage disequilibrium analysis

To detect LD decay in occidental and oriental barley sub-populations, correlation coefficient (*r*^2^) values were calculated using Haploview v. 4.2^64^ with the setting -dprime -nogui. Average *r*^2^ values for each physical distance were calculated using original Perl scripts. LD blocks in occidental and oriental barley sub-populations were found using Haploview with the setting -blockoutput GAB -nogui. The LD blocks found were superimposed on the Morex reference genome using circos.

## Additional Information

**Accession codes**: The read data have been submitted to DDBJ Sequence Read Archive [DDBJ: DRA003931].

**How to cite this article**: Takahagi, K. *et al*. Analysis of single nucleotide polymorphisms based on RNA sequencing data of diverse bio-geographical accessions in barley. *Sci. Rep.* **6**, 33199; doi: 10.1038/srep33199 (2016).

## Supplementary Material

Supplementary Information

## Figures and Tables

**Table 1:** Summary of the RNA-seq results for the 20 barley accessions.

Species	Accession	Total No. of reads	Total No. of mapped reads	Percent of mapped reads	Average reads length (bp)
*H. spontaneum*	H602	10,022,606	9,358,952	93.38%	136
*H. vulgare*	B669	15,479,737	14,721,023	95.10%	125
	C319	15,144,777	14,487,337	95.66%	145
	C346	15,176,183	14,345,822	94.53%	146
	C656	16,026,087	15,406,069	96.13%	145
	E245	15,341,723	14,679,342	95.68%	149
	E612	11,201,259	10,323,600	92.16%	145
	I304	9,081,490	8,402,871	92.53%	145
	I335	13,541,901	12,925,132	95.45%	164
	I622	16,406,298	15,659,799	95.45%	152
	I626	14,872,103	14,176,640	95.32%	161
	J064	15,403,340	14,384,534	93.39%	152
	J247	9,845,797	8,962,223	91.03%	150
	J647	10,068,027	9,195,339	91.33%	145
	K692	15,788,289	15,112,668	95.72%	124
	K735	11,214,410	10,408,421	92.81%	160
	N009	16,761,966	15,927,479	95.02%	143
	T567	15,294,760	14,574,021	95.29%	142
	U051	15,711,210	15,027,237	95.65%	141
	U353	15,519,320	14,754,353	95.07%	160
Total		277,901,283	262,832,862	94.33%	147

**Table 2:** Number of RNA-seq based SNPs called by the various tools in comparison with the genomic framework of barley.

Species	Accession	No. of SNPs			
		freebayes	glfMultiples	samtools	fgs*
*H. spontaneum*	H602	67,462	147,417	91,442	55,403
*H. vulgare*	B669	64,472	210,241	108,105	49,094
	C319	100,387	257,743	150,167	79,903
	C346	99,545	262,354	149,535	78,364
	C656	100,366	257,088	148,563	77,253
	E245	90,395	241,736	137,109	69,761
	E612	62,054	155,797	88,866	49,325
	I304	50,712	119,733	71,578	41,676
	I335	85,453	229,912	131,492	66,382
	I622	102,344	262,084	152,592	79,949
	I626	92,970	243,682	141,367	72,405
	J064	75,308	161,268	101,226	59,153
	J247	51,806	130,889	73,312	38,729
	J647	63,195	148,909	89,326	51,591
	K692	93,428	242,556	139,799	75,444
	K735	54,103	134,995	78,892	41,098
	N009	95,951	244,587	144,688	75,198
	T567	72,187	211,850	114,637	55,206
	U051	83,207	230,728	128,448	64,319
	U353	78,417	213,824	119,391	59,136

^*^Intersection of the identified SNPs using the three SNP callers: freebayes, glfMultiples, and samtools.

**Table 3:** Average haplotype diversity and average pairwise *F*_*ST*_ between each sub-population of domesticated barley.

Sub-populations	haplotype diversity	pairwise *F*_*ST*_		
		Occidental	Oriental	Marginal
Occidental	0.68			
Oriental	0.73	0.33		
Marginal	0.81	0.12	0.19	
