# Supplementary material for: Analysis of single nucleotide polymorphisms based on RNA sequencing data of diverse bio-geographical accessions in barley
Source: Sci Rep. 2016 Sep 12;6:33199. doi: 10.1038/srep33199 (PMC5018957; doi:10.1038/srep33199)

SUPPLEMENTARY INFORMATION

Analysis of single nucleotide polymorphisms based on RNA sequencing data of diverse bio-geographical accessions in barley

Kotaro Takahagi, Yukiko Uehara-Yamaguchi, Takuhiro Yoshida, Tetsuya Sakurai, Kazuo Shinozaki, Keiichi Mochdia and Daisuke Saisho

Table S1. List of barley accessions used for RNA sequencing in this study.

Table S2. No. of exome-sequencing-based SNPs called various tools in comparison with the genomic framework of barley.

Table S3. No. of transcribed regions with sub-population specific SNPs in barley.

Figure S1. Number of novel transcribed regions matching grass proteins. Number of putative novel transcribed regions in barley that showed high sequence similarity with genes annotated for the genome of grass species (left bars), and number of protein sequences showing homology with queried barley sequences (right bars).

Figure S2. Delta K plots in STRUCTURE analysis. Delta K values calculated in STRUCTURE analysis using the bi-allelic SNP dataset derived from the 31 domesticated barley accessions, plotted against each K.

Table S1. List of barley accessions used for RNA sequencing in this study.

OU No	Taxon	Name	Origin [a]	caryopsis	kernel rows	Vernalization requirement
H602	Wild	var. transcaspicum	Caspian Sea region	+	2	3
B669	Landrace	Suez (84)	Egypt	+	6	1
C319	Landrace	Chihchou	Annwei, China	+	6	4
C346	Landrace	Shanghai 1	Kiangsu, China	n	6	4
C656	Landrace	Tibet White 4	Tibet, China	n	6	1
E245	Landrace	Addis Ababa 40 (12-24-84)	Ethiopia	+	L	1
E612	Landrace	Ethiopia 36 (CI 2225)	Ethiopia	+	D	1
I304	Landrace	Rewari	India	+	6	1
I335	Landrace	Ghazvin 1 (184)	Iran	+	2	1
I622	Landrace	H.E.S. 4 (Type 12)	Afghanistan	+	6	1
I626	Landrace	Katana 1 (182)	Syria	+	2	3
J064	Landrace	Hayakiso 2	Shimane, Japan	+	6	4
J247	Improved variety	Haruna Nijo	Tochigi Pref. AES, Japan	+	2	1
J647	Landrace	Akashinriki	Shizuoka, Japan	n	6	2
K692	Landrace	Eumseong Covered 3	Chungcheongbug, Korea	+	6	5
K735	Landrace	Natsudaikon Mugi	Suweon AES, Korea	+	6	1
N009	Landrace	Tilman Camp 1 (1398)	Nepal	n	6	3
T567	Landrace	Goenen (997)	Turkey	+	6	1
U051	Landrace	Archer	United Kingdom	+	2	1
U353	Landrace	Opal	Denmark	+	2	1

a. Information of origins are provided by BarleyDB (https://shigen.nig.ac.jp/barley/).

Table S2. No. of exome-sequencing-based SNPs called various tools in comparison with the genomic framework of barley.

Species	Accession	No. of SNPs			
		freebayes	glfMultiples	samtools	fgs*
H. spontaneum	B1K-03-07	1,584,583	2,080,999	2,040,449	1,565,199
	B1K-04-12	383,259	506,205	494,617	378,397
	H602**	361,087	474,634	463,521	355,980
H. vulgare	Barke	2,304,320	2,907,232	2,863,705	2,284,239
	Bonus	296,520	403,174	390,078	288,775
	Borwina	351,857	501,180	486,562	344,561
	Bowman	560,103	778,128	752,970	544,542
	Foma	328,687	450,463	434,992	319,256
	Gull	291,264	397,920	384,709	283,277
	Harrington	264,985	364,969	355,202	260,004
	Haruna Nijo**	273,015	371,076	360,915	268,235
	Igri	298,010	411,962	400,697	292,377
	Kindred	189,495	274,279	265,556	184,824
	Steptoe	666,103	894,098	865,450	647,121
	Vogelsanger Gold	357,436	501,753	487,306	350,157

*Intersection of identified SNPs using 3 SNP callers; freebayes, glfMultiples and samtools.

**SNPs data of these accessions were used only for the assesment of the accuracy of the RNA-seq-based SNPs.

Table S3. No. of transcribed regions with sub-population specific SNPs in barley.

		No.
SNPs sub-populatins specific SNPs*		1804
Transcribed regions with sub-population specific SNPs	Annotated genes	982
	Putative novel transcrived regions	188

*SNPs with >= 75% major allele (A) in oriental accessions and with >= 75% major allele (B) in occidental accessions.

Figure S1

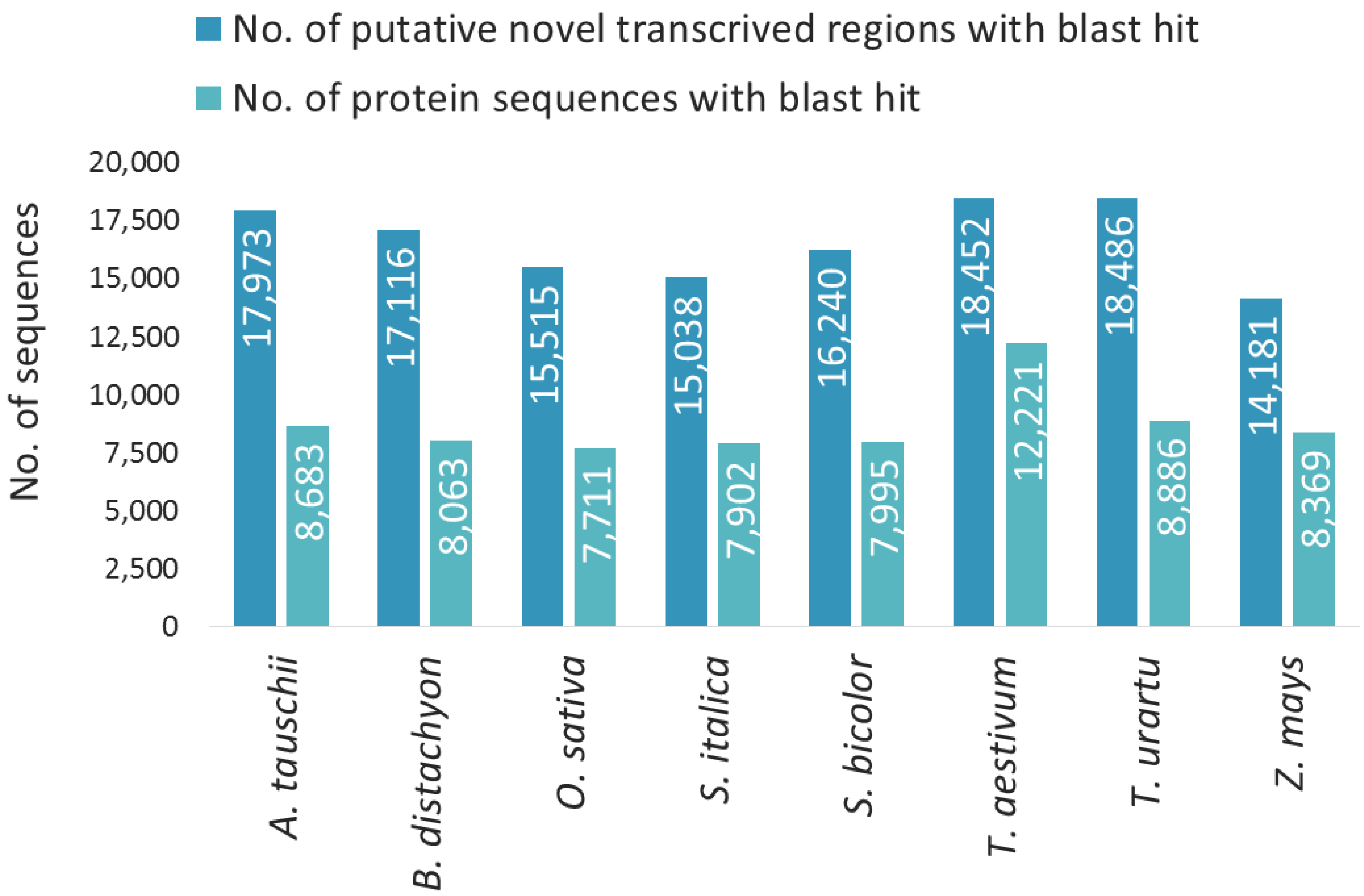
No. of putative novel transcrived regions with blast hit
No. of protein sequences with blast hit
No. of sequences
20,000
17,500
15,000
12,500
10,000
7,500
5,000
2,500
0
17,973
8,683
17,116
8,063
15,515
7,711
15,038
7,902
16,240
7,995
18,452
12,221
18,486
8,886
14,181
8,369
A. tauschii
B. distachyon
O. sativa
S. italica
S. bicolor
T. aestivum
T. urartu
Z. mays

Figure S2

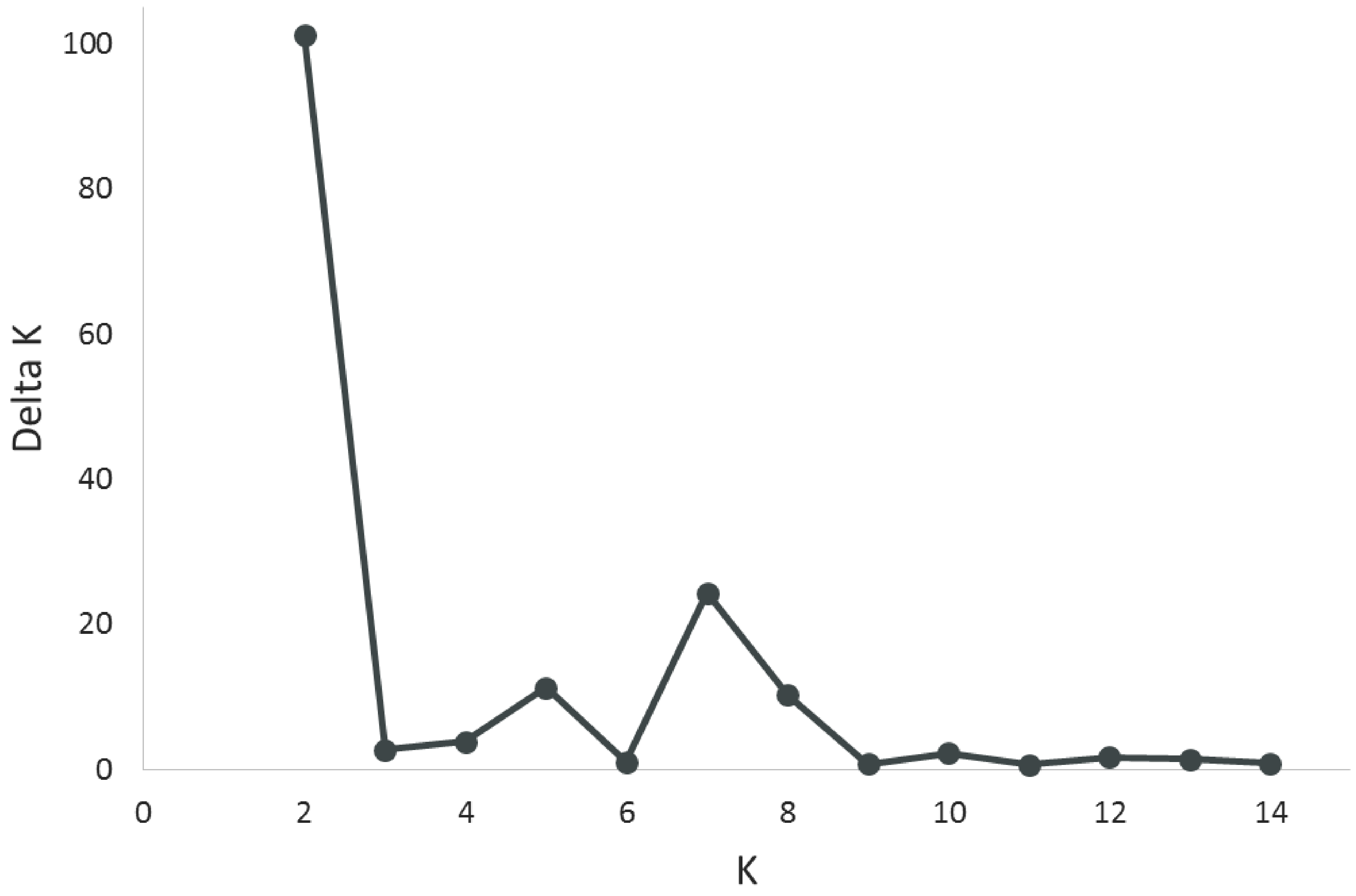

Supplement: Supplementary Information [file srep33199-s1.pdf]
